# Plasma and urinary extracellular vesicles as a source of RNA biomarkers for prostate cancer in liquid biopsies

**DOI:** 10.3389/fmolb.2023.980433

**Published:** 2023-02-03

**Authors:** Cristina Bajo-Santos, Agnese Brokāne, Pawel Zayakin, Edgars Endzeliņš, Kristīne Soboļevska, Alberts Belovs, Juris Jansons, Māris Sperga, Alicia Llorente, Ilze Radoviča-Spalviņa, Vilnis Lietuvietis, Aija Linē

**Affiliations:** ^1^ Latvian Biomedical Research and Study Centre, Riga, Latvia; ^2^ Riga Stradiņš University, Riga, Latvia; ^3^ Department Molecular Cell Biology, Institute for Cancer Research, Oslo University Hospital, Oslo, Norway; ^4^ Department for Mechanical, Electronics and Chemical Engineering, Oslo Metropolitan University, Oslo, Norway; ^5^ Genera Ltd., Riga, Latvia

**Keywords:** extracellular vesicles, liquid biopsy, RNA biotypes, RNA sequencing, digital droplet PCR, prostate cancer

## Abstract

**Introduction:** Extracellular vesicles (EVs) have emerged as a very attractive source of cancer- derived RNA biomarkers for the early detection, prognosis and monitoring of various cancers, including prostate cancer (PC). However, biofluids contain a mixture of EVs released from a variety of tissues and the fraction of total EVs that are derived from PC tissue is not known. Moreover, the optimal biofluid—plasma or urine—that is more suitable for the detection of EV- enclosed RNA biomarkers is not yet clear.

**Methodology:** In the current study, we performed RNA sequencing analysis of plasma and urinary EVs collected before and after radical prostatectomy, and matched tumor and normal prostate tissues of 10 patients with prostate cancer.

**Results and Discussion:** The most abundant RNA biotypes in EVs were miRNA, piRNA, tRNA, lncRNA, rRNA and mRNA. To identify putative cancer-derived RNA biomarkers, we searched for RNAs that were overexpressed in tumor as compared to normal tissues, present in the pre-operation EVs and decreased in the post-operation EVs in each RNA biotype. The levels of 63 mRNAs, 3 lncRNAs, 2 miRNAs and 1 piRNA were significantly increased in the tumors and decreased in the post-operation urinary EVs, thus suggesting that these RNAs mainly originate from PC tissue. No such RNA biomarkers were identified in plasma EVs. This suggests that the fraction of PC-derived EVs in urine is larger than in plasma and allows the detection and tracking of PC-derived RNAs.

## Introduction

Prostate cancer (PC) is the second most common cancer in males affecting more than 1.27 million men per year worldwide (Culp et al., 2020). It is a highly heterogeneous disease—some patients develop a high-grade disease with extracapsular spread or distant metastases requiring aggressive treatment, while others have a relatively indolent, slowly progressing disease that can be managed by active surveillance (AS) (Fitzpatrick et al., 2014; Romero-Otero et al., 2016). However, predicting which patients require treatment and which can be safely managed by AS is still a major challenge for urologists and oncologists (Overland et al., 2019). Unlike the majority of other solid cancers, PC can be detected using a simple blood test–prostate specific antigen (PSA) test. Introduction of PSA test has substantially improved early detection of PC. However, elevated serum PSA levels are also found in patients with benign prostatic hyperplasia (BPH) and prostatitis (Zackrisson et al., 2003; Kawakami et al., 2004; Agnihotri et al., 2014), as well as in healthy males after ejaculation, prostate biopsy and exercise (Mejak et al., 2013). In fact, some studies have shown that only 22%–26% of men with elevated PSA levels (4.0–9.9 ng/mL) have cancer, hence PSA testing leads to large number of unnecessary prostate biopsies and emotional morbidity (Catalona et al., 1991; Brawer et al., 1992; Yamamoto et al., 2020; Bennett et al., 2022). At the same time, the false negative rate of PSA test is about 15% (Thompson et al., 2004). Moreover, the PSA test is unable to discriminate between aggressive and clinically insignificant disease and therefore has led to overdiagnosis and subsequent overtreatment of patients with an indolent disease (Andriole et al., 2012; Schröder et al., 2012).

Liquid biopsies are samples of biofluids that are used for the analysis of cancer cells or cancer tissue-derived molecules (Babayan and Pantel, 2018). Liquid biopsies have emerged as a promising alternative to conventional tissue biopsies since they can be obtained in a non-invasive or minimally invasive way, thus avoiding the risks related to the collection of tissue biopsies and allowing serial sampling during the course of disease. In PC, they have a potential utility for diagnosis, differentiating between aggressive and indolent PC, active surveillance, post-operative monitoring, early detection of recurrence and tracking tumor evolution. The most common analytes in liquid biopsies are circulating tumor cells and circulating cell-free DNA or RNA (Pantel and Alix-Panabières, 2019; Siravegna et al., 2019). More recently, extracellular vesicles (EVs) have been proposed as an alternative source of biomarkers in liquid biopsies.

The term “EV” refers to all types of membrane-bound vesicles released from cells in the extracellular space (Théry et al., 2018). The main types of EVs are exosomes, microvesicles (also called ectosomes, shedding vesicles or microparticles) and apoptotic bodies that are generated by different biogenetic pathways and differ in their molecular content and functions in the body (Yáñez-Mó et al., 2015; van Niel et al., 2018). EVs contain various lipids, proteins, metabolites, RNAs including protein coding and non-coding RNAs such as long non-coding RNAs (lncRNAs), microRNAs (miRNAs) and PIWI-interacting RNAs (piRNAs), and even DNA fragments (Yáñez-Mó et al., 2015; Movahedpour et al., 2022). Overall, molecular cargo of EVs is reminiscent of their cell of origin, however various sorting mechanisms exist that can sort selective cargo into EV lumen thus leading to the depletion or enrichment of some molecules in EVs (Vasconcelos et al., 2019; Seyedaghamiri et al., 2022). EVs are released by virtually all cell types in the body and are present in various body fluids (Murillo et al., 2019). EVs isolated from biofluids of cancer patients have been found to contain cancer-associated miRNA signatures (Broggi et al., 2019; García-Silva et al., 2019) and mRNA fragments carrying cancer-specific mutations (Bao et al., 2018; Yap et al., 2020; Pasini et al., 2021), suggesting that the EV RNA cargo may be used for the detection and monitoring of cancer. In PC patients, cancer-derived EVs are released into the blood and urine, but the fraction of cancer-derived EVs in these biofluids is currently unknown (Ramirez-Garrastacho et al., 2022a), and to the best of our knowledge, a direct comparison of RNA cargo in plasma and urinary EVs has not been reported so far. In the current study, we performed RNA sequencing analysis of plasma and urinary EVs collected before and after radical prostatectomy, and matched tumor and normal prostate tissues of 10 PC patients. To identify putative cancer-derived RNA biomarkers, we searched for RNAs that were overexpressed in tumor as compared to normal tissues (LogFC>1, adj. *p* < 0.05), present in the pre-operation EVs (at least 10 raw reads in one of the samples) and decreased in the post-operation EVs (LogFC>1, adj. *p* < 0.05) in each RNA biotype.

## Materials and methods

### Patients and sample processing

A total of 30 patients with newly diagnosed resectable PC were enrolled in this study between October 2018 and January 2020 at Riga East University Hospital and were followed-up until September 2021. All patients had elevated levels of PSA (2.5–50 ng/mL) at the time of diagnosis. Patient exclusion criteria included: blood transfusion in the last 6 months, another oncological disease, urinary tract infection and use of long-term urinary catheter. Clinical characteristics of the study population are provided in Table 1.

**TABLE 1:** Patient characteristics.

		Discovery cohort (RNA-seq)		Validation cohort (RT-ddPCR)	
		PC		PC	
Number		10		20	
Age (Median, years)		66.4		65.9	
Age (range)		60–73		49–74	
Diagnostic PSA (ng/mL)		Number	%	Number	%
	<4	0	0	2	10
	4–10	7	70	12	60
	>10	3	30	6	30
PostOp PSA (ng/mL)					
	<1	9	90	18	90
	>1–4	0	0	2	10
	>10	1	10	0	0
Gleason Score					
	6	1	10	10	50
	7 (3 + 4)	7	70	3	15
	7 (4 + 3)	1	10	3	15
	8	1	10	3	15
	9	0	0	1	5
Clinical T-Staging					
	T2a	3	30	6	30
	T2b	2	20	7	35
	T3a	3	30	5	25
	T3b	2	20	2	10

Sixty ml of the first morning urine were collected, centrifuged at 2000 g for 15 min at room temperature, aliquoted and stored at −80°C. Blood samples were collected in EDTA-coated tubes and processed at room temperature within 2 h. Plasma samples were centrifuged twice at 3000 g for 10 min, aliquoted and stored at −80°C. Samples were collected at two different time points: before radical prostatectomy (PreOp) and 3 months after the surgery (PostOp).

Tumor and normal prostate tissue samples were macroscopically dissected immediately after the surgery by an experienced uropathologist. One slice of the tissue specimens was subjected to histological evaluation in order to verify the presence or absence of tumor cells in the tissue specimens and to assess the Gleason score in the given specimen, whereas the other part of the specimen was immediately placed into the RNALater solution (Thermo Fisher Scientific, United States) and stored at −20°C until processing.

The study was conducted according to the Declaration of Helsinki. The specimens were collected after the patients’ informed written consent was obtained and anonymized. The study protocol was approved by the Latvian Central Medical Ethics Committee (decision No. 01-29.1/488).

### Isolation of extracellular vesicles

EVs were isolated from the plasma and urine samples using size exclusion chromatography (SEC) as described before (Endzeliņš et al., 2017) with some modifications. Briefly, the urine samples (20 mL) were thawed at +37°C in a water bath and centrifuged at 10 000 g for 15 min at + 4°C to remove large vesicles and some uromodulin and concentrated up to 500 µL using 100 kDa centrifugal filters (Merck Millipore, United States). The concentrated urine samples and 1 mL of plasma were loaded on Sepharose CL2B 10 mL columns. The eluate was collected in 12 sequential 0.5 mL fractions and each fraction was measured with Zetasizer Nano ZS (Malvern, United Kingdom). Fractions containing particles larger than 30 nm were combined and concentrated up to 100 μL using 3 kDa centrifugal filters (Merck Millipore, United States). EV samples were treated with Proteinase K (1 mg/mL) (Thermo Fisher Scientific) for 60 min at 37°C followed by RNAse A (100 ng/μL) (Thermo Fisher Scientific) treatment for 15 min at 37°C. The purity, size distribution profile and concentration of EVs were assessed by transmission electron microscopy (TEM) and nanoparticle tracking analysis (NTA) using NanoSight NS500 instrument (Malvern, United Kingdom) as described before (Endzeliņš et al., 2017).

### Western Blot

EVs were lysed in RIPA buffer (50 mM Tris, pH 8.0, 150 mL NaCl, 1% Triton X-100, 0.5% Na deoxycholate, 0.1% SDS). Protein concentration was measured using PierceTM BCA Protein Assay Kit (Thermo Fisher Scientific). LNCaP cells (ATCC, Manassas, VA, United States) were used as a positive control. Equal fraction (one fifth) of the EV proteins and 10 µg of cellular proteins were separated by 10% SDS-PAGE, transferred to nitrocellulose membranes and blocked using 10% (w/v) fat-free milk. Membranes were incubated with primary antibodies against TSG101 (Abcam, #ab15011, 1:1000 dilution), Calnexin (Abcam, #ab22595, 1:2000 dilution), CD63 (Santa Cruz Biotechnology, #sc-5275, 1:500 dilution) and PDCD6IP/ALIX (Santa Cruz Biotechnology, # sc-166952, 1:1000 dilution). Membranes were washed and incubated with horseradish peroxidase-conjugated goat anti-rabbit IgG, F(ab’)2-HRP: (Santa Cruz Biotechnology, #sc-3837) or goat anti-mouse m-IgGκ BP-HRP (Santa Cruz Biotechnology, #sc-516102) in 1:2000 dilution. Immunoreactive bands were visualized using Western Blotting Detection Reagent kit (GE Healthcare Lifesciences) and pictures were taking using a Nikon d610 dSLR body (Nikon) with Sigma 35 mm f/1.4 DG HSM Art lens (Sigma).

### RNA isolation and sequencing

Approximately 20 mg of tissues were homogenized using QIAzol Lysis Reagent (Qiagen, United States) and Lysing Matrix A tubes in a FastPrep-24 homogenizer (MP Biomedicals, United States). RNA was isolated using miRNeasy Micro Kit (Qiagen) following the small RNA enrichment protocol provided by the manufacturer, thus obtaining both, the long and small RNA fraction of each sample. From EVs, RNA was extracted using miRNeasy Micro Kit. On column DNAse treatment was performed according to the manufacturer’s instructions. RNA quantity and quality was assessed using Agilent pico RNA kit and Agilent 2100 Bioanalyzer (Agilent technologies, United States).

EV-RNA libraries were constructed from half of the entire yield of extracted EV RNA without any size separation steps, whereas tissue small RNA libraries were constructed from 10 ng of tissue small RNA fraction using CleanTag^®^ Small RNA Library Prep Kit (Trilink Biotechnologies, United States). The libraries were cleaned using Blue Pippin DNA Size Selection with 3% gel Blue Pippin Cassette (Sage Science, United States) setting target length to 130–250 bp. All libraries were constructed in duplicates. The libraries were sequenced on Illumina NextSeq500 instrument using NextSeq 500/550 Mid Output Kit v2.5 (150 cycles) (Illumina, United States).

Transcriptome libraries were built in duplicates from 100 ng of long tissue RNA fraction using TruSeq Stranded mRNA library Prep (Illumina, United States) following manufacturer’s instructions. Libraries were size-selected using Blue Pippin system with 2% gel Blue Pippin Cassette (Sage Science, United States) with a range of 200–600 bp and their quality and quantity was assessed using Agilent DNA kit (Agilent technologies, United States). Libraries were pooled and sequenced using a NextSeq 500/550 High Output Kit v2.5 (300 cycles) (Illumina, United States).

### RNA sequencing data analysis

The obtained raw data in FASTQ format were analyzed using ad-hoc R script pipeline. For small RNA libraries, it included the trimming of adapters using Cutadapt (Martin, 2011), read mapping against Ensembl human genome (GRCh38) using Bowtie2 (Langmead and Salzberg, 2012), repositioning of multi-aligned reads using ShortStack (Axtell, 2013), counting using htseq-count package (Putri et al., 2022) with GRCh38 and miRbase (Kozomara et al., 2019), GtRNAdb (Chan and Lowe, 2016), LNCipedia (Volders et al., 2018), lncRNAdb (Quek et al., 2015), piRBase (Wang et al., 2018), piRNABank (Sai Lakshmi and Agrawal, 2008) and piRNAdb (Piuco and Galante, 2021) annotations. For transcriptome libraries, reads were mapped using STAR (Dobin et al., 2012) and only unique alignments were counted. For differentially expressed gene (DEG) analysis, the reads were normalized and analyzed using DESeq2 package (Love et al., 2014). Multiple testing correction was done by the Benjamini-Hochberg procedure and adjusted (adj.) *p*-value of ≤0.05 was considered to be significant. The RNAseq datasets are available in ArrayExpress, accession No. E-MTAB-11910.

### Reverse transcription—droplet digital PCR

A half of the entire yield of extracted EV RNA was reverse-transcribed using miRCURY LNA RT kit (Qiagen) according to the manufacturer’s protocol. A total of 20 µL of PCR reaction containing 1:2 diluted cDNA, 10 µL of 2xEvaGreen Supermix (Bio-Rad) and either 1 µL of miRCURY LNA primer mix (Qiagen) or 2 µL of QuantiNova LNA primer mix (Qiagen) (Supplementary Table S1) was loaded into a disposable droplet generator cartridge (Bio-Rad). Then, 70 µL of droplet generation oil for EvaGreen was loaded in the corresponding wells and placed into a QX200 droplet generator (Bio-Rad). Once droplets were generated, they were transferred to a ddPCR clear semi-skirted 96-well plate (Bio-Rad), covered with a Pierceable Foil Heat Seal (Bio-Rad) and amplified in a T100 Thermal Cycler (Bio-Rad) under the following conditions: 95°C for 5 min; 40 cycles at 95°C for 30 s followed by specific primer annealing temperature (Supplementary Table S1); 4°C for 5 min; 90°C for 5 min and indefinite hold at 4°C. Programme was run at 2°C/sec rampage rate. Plate was read using a QX200 Droplet Reader (Bio-Rad) and results were analyzed using QuantaSoft™ Software (Bio-Rad). The optimal annealing temperature was determined for each assay by running it across a thermal gradient (50°C–60°C).

### Statistical analysis

Statistical analyses were performed using GraphPad Prism 6.2 (GraphPad, United States). Comparison between PreOp vs. PostOp data was assessed using Wilcoxon matched-paired signed rank test. Kruskal–Wallis test with multiple comparisons corrected by Dunn’s test was performed to determine differences among RNA biotypes. *p* value ≤0.05 was considered significant.

## Results

### Yield, size and purity of EVs

**FIGURE 1:**
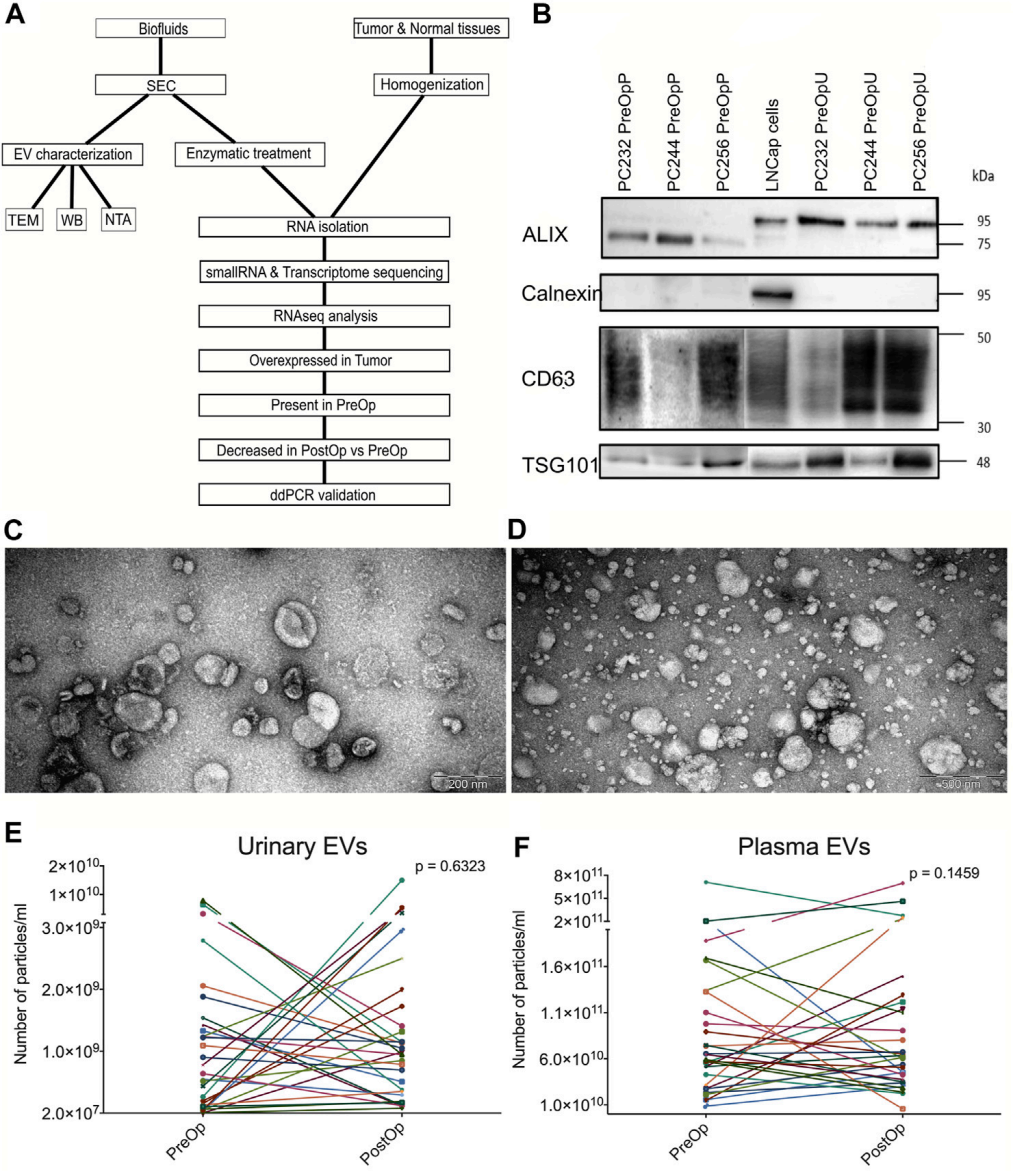
EV isolation and characterization. **(A)** Workflow of the study. **(B)** Western blot of CD63, ALIX, TSG101 and calnexin in LNCaP prostate cancer cells and pre-operation urinary and plasma EVs from three different patients. **(C)** Representative TEM image of urinary EVs. **(D)** Representative TEM image of plasma EVs. **(E)** Paired dot plots showing the numbers of EVs per ml of urine before (PreOp) and after radical prostatectomy (PostOp). **(F)** Paired dot plots showing the numbers of EVs per ml of plasma before and after radical prostatectomy. Wilcoxon matched-paired signed rank test was used to assess the statistical significance of the differences between groups.

The workflow of this study is shown in Figure 1A. EVs were isolated from matched plasma and urine samples of PC patients collected before radical prostatectomy (RP) and at 3 months after the surgery. In order to evaluate the purity and size distribution of EVs, the obtained EVs were characterized using transmission electron microscopy (TEM) and Western blot (WB) analysis (Figures 1B–D). WB results showed that EVs were positive for ALIX (official symbol PDCD61P), TSG101 and CD63, which are characteristic EV markers (Théry et al., 2018). In plasma EVs, the molecular weight of ALIX is ∼75 kDa that corresponds to the C-terminal proteolytic cleavage product (Vanessa et al., 2019). EVs were negative for calnexin, an endoplasmic reticulum protein (Figure 1B), thus showing that the EV preparations do not contain significant contamination of ER membranes. TEM revealed that the majority of particles in urine samples were ranging in size from 30 to 150 nm and had a cup-shaped morphology that is typically observed for exosomes using this TEM protocol (Figure 1C). In plasma samples, EVs were ranging in size from 30 to 250 nm, but smaller particles (<30 nm in diameter) were also present (Figure 1D). In order to assess the yield and EV dynamics before and after the RP, EVs were evaluated by nanoparticle tracking analysis (NTA) (Figures 1E, F). Results showed that the number of particles per mL of urine (Figure 1E) ranged from 2.26 × 10^7^ to 1.5 × 10^10^ particles/mL while in plasma it ranged from 5.68 ×10^9^ to 7.10 ×10^11^ particles per ml (Figure 1F). The EV yields are consistent with those reported in other studies (Li et al., 2014; Endzeliņš et al., 2017) and no significant difference was found between EV numbers before and after RP (Figures 1E, F).

### Composition of EV RNA cargo

To characterize the composition of RNA cargo in plasma and urinary EVs, we performed deep sequencing of RNA libraries constructed from the following bio-specimens from 10 PC patients: pre-operation plasma (PreOpP), 3 months post-operation plasma (PostOpP), pre-operation urine (PreOpU), 3 months post-operation urine (PostOpU), and small RNA fractions of histologically verified PC tissue (T_S_) and normal prostate tissue (N_S_). Moreover, we reasoned that only fragments of degraded mRNAs and lncRNAs are present in small RNA libraries constructed from tissue specimens. Therefore, in order to obtain unbiased long RNA expression profiles, we also built full transcriptome libraries from tumor (T_L_) and normal prostate (N_L_) tissues.

**FIGURE 2:**
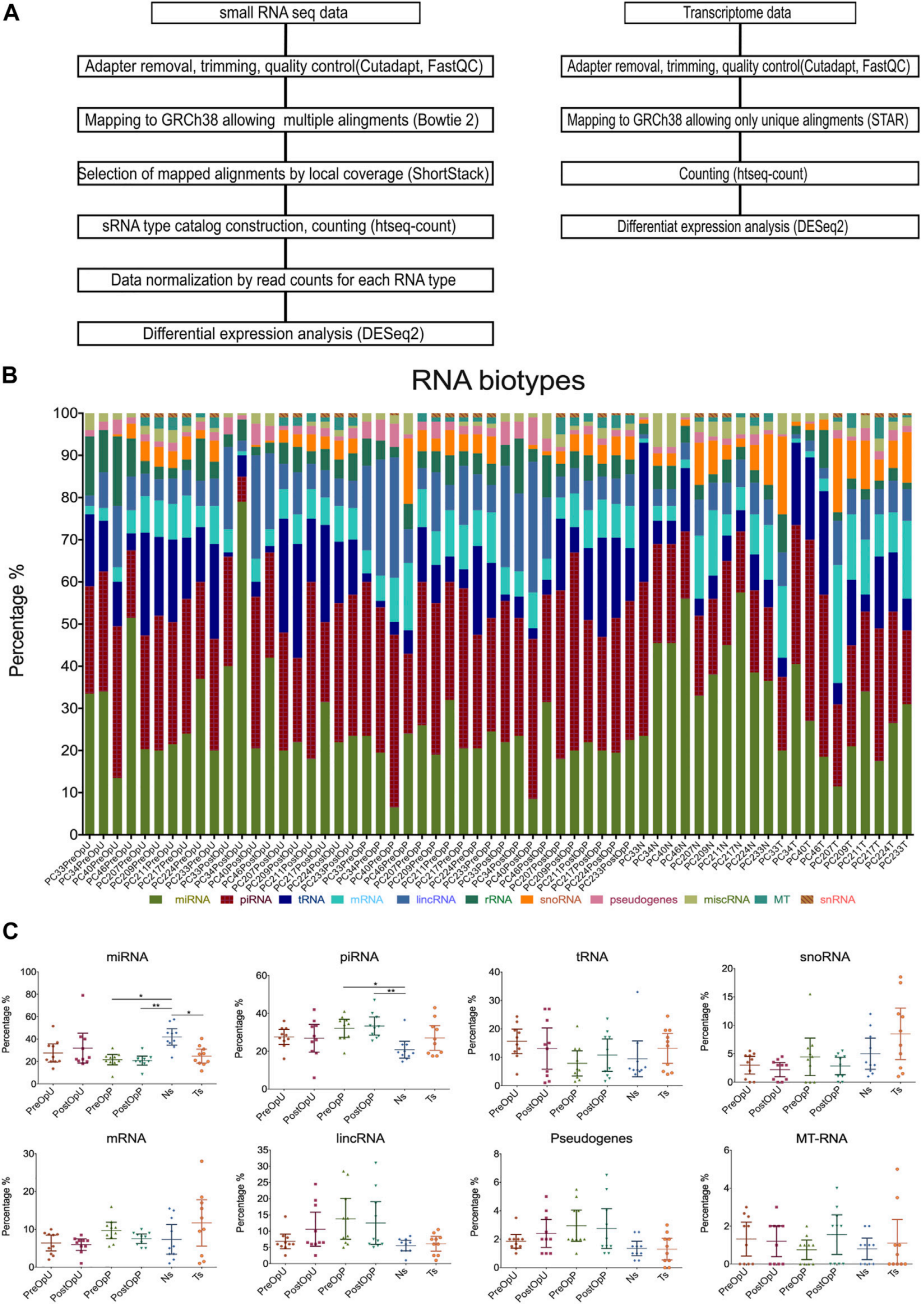
RNA biotypes. **(A)** RNA sequencing data analysis pipeline. **(B)** Percentage of reads representing various RNA biotypes in each sample. **(C)** Dot plots showing the comparison of specific RNA biotypes in the groups of samples. Kruskal—Wallis test with multiple comparisons corrected by Dunn’s test was used to assess the statistical significance of the differences between groups. *p < 0.5; **p < 0.01; ***p < 0.001. PreOpU—urinary EVs before radical prostatectomy, PostOpU—urinary EVs after radical prostatectomy; PreOpP—plasma EVs before radical prostatectomy, PostOpP—plasma EVs after radical prostatectomy, N—normal prostate tissue, T—tumor tissue.

We have shown before that more than 50% of the EV-associated RNA is attached to the surface of EVs (Endzeliņš et al., 2017). In the current study, we focused on the intraluminal RNAs, therefore EVs were treated with proteinase K and RNAse A prior to the RNA extraction. On average a total of 4.45 million raw reads were obtained per sample, and an average of 3.38 million reads remained after quality control, adaptor trimming and filtering out fragments smaller than 18 nt. To assess the representation of various RNA biotypes in plasma and urinary EVs, the reads mapped to overlapping features in human genome were prioritized in the following order: miRNAs > tRNAs > rRNA > mRNAs > pseudogenes > snRNAs > snoRNAs > piRNAs > lncRNAs > miscRNAs. The data analysis pipeline is shown in Figure 2A. The percentage of reads corresponding to each RNA biotype is shown in Figure 2B. In average, the RNA cargo of urinary EVs predominantly comprised miRNAs (32%), piRNAs (26.5%) and tRNAs (15%) followed by lincRNA (8%), rRNAs (6%) and fragments of mRNAs (5.5%), while plasma EVs contained higher proportion of piRNAs (32.5%), followed by miRNAs (21%), lincRNAs (13%), tRNAs (9.5%) and fragments of mRNAs (8.5%) (Figure 2B). Plasma EVs had higher piRNA and lower miRNA fraction compared to normal prostate tissues. No statistically significant differences in RNA biotypes composition were observed between PreOp and PostOp EV samples (Figure 2C).

### Identification of prostate cancer-derived EV-enclosed RNAs

**FIGURE 3:**
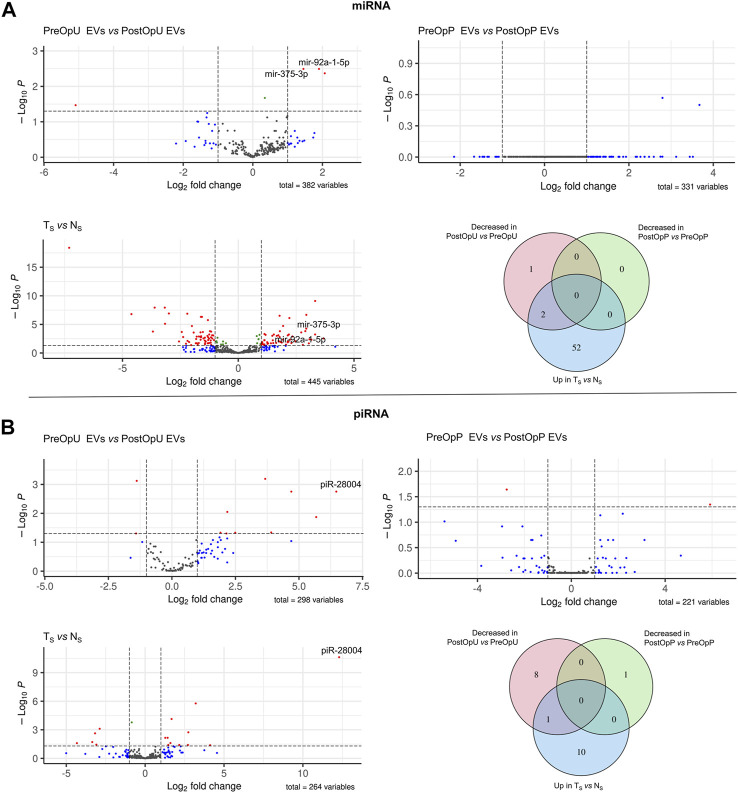
Differential expression analysis of small RNAs. **(A)** Differential expression analysis of miRNAs and **(B)** piRNAs. Volcano plots depict significant differences in the pre-operation urinary or plasma EVs as compared to the post-operation EVs, and in small RNA libraries prepared from tumor (T_S_) and normal prostate tissues (N_S_). Venn diagrams show the numbers of small RNAs overexpressed in tumor tissues vs. normal prostate tissues (Up in T_S_ vs. N_S_), decreased in the post-operation urinary EVs as compared to pre-operation urinary EVs and decreased in the post-operation plasma EVs as compared to pre-operation plasma EVs (Log2FC >1 and adj.*p*-value ≤0.05).

**FIGURE 4:**
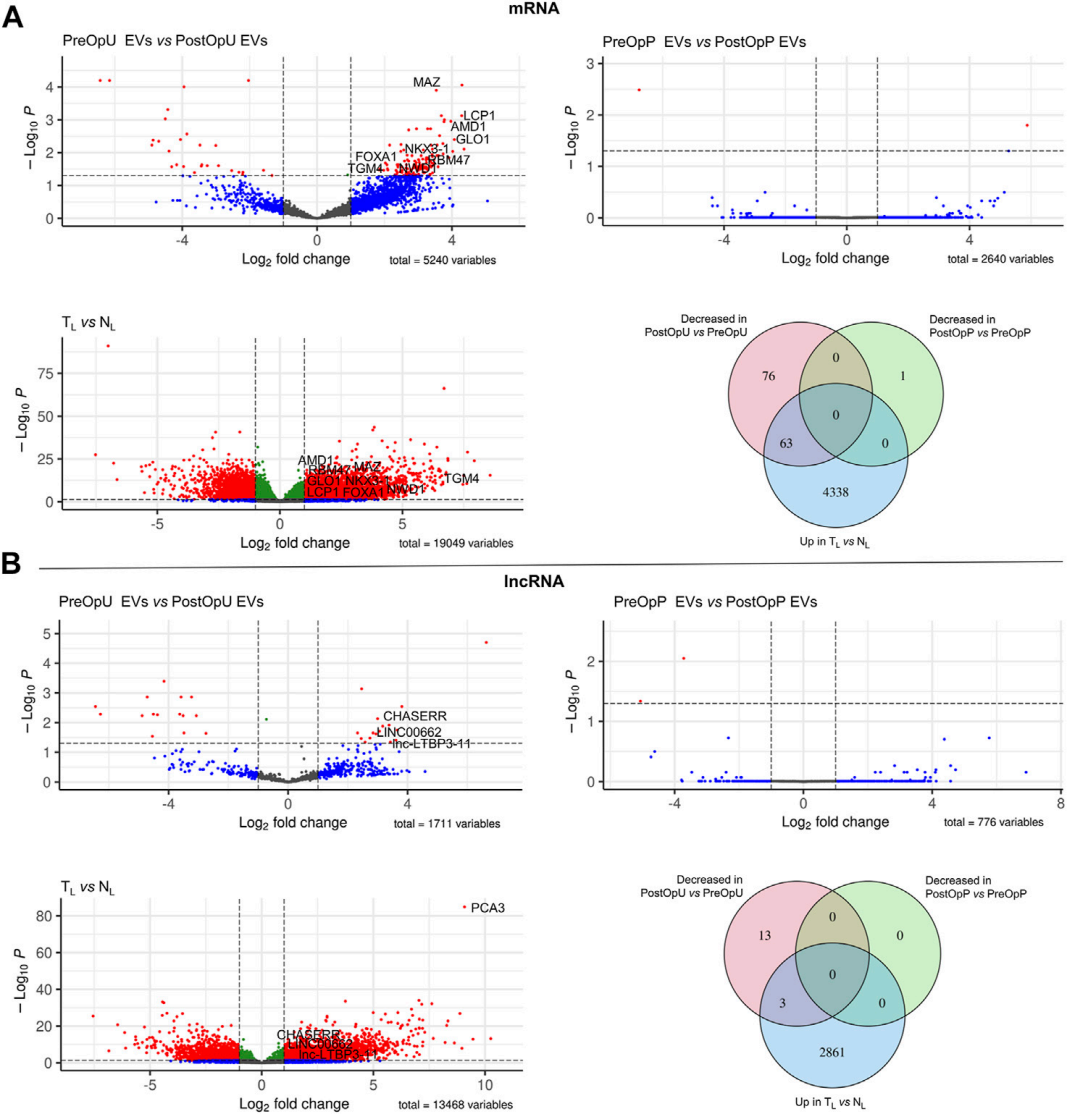
Differential expression analysis of long RNAs. **(A)** Differential expression analysis of mRNAs and **(B)** lncRNAs. Volcano plots depict significant differences in the pre-operation urinary or plasma EVs as compared to the post-operation EVs, and in full transcriptome libraries prepared from tumor (T_L_) and normal prostate tissues (N_L_). Venn diagrams show the numbers of mRNAs and lncRNAs overexpressed in tumor tissues vs. normal prostate tissues (Up in T_L_ vs. N_L_), decreased in the post-operation urinary EVs as compared to pre-operation urinary EVs and decreased in the post-operation plasma EVs as compared to pre-operation plasma EVs (Log2FC >1 and adj.*p*-value ≤0.05).

To identify EV-enclosed RNAs that are derived from PC tissues, we searched for RNAs that met the following criteria in each RNA biotype separately: (1) overexpressed in tumor tissues as compared to normal prostate tissues (LogFC>1, adj. *p* < 0.05); (2) present in the PreOp EVs (at least 10 raw reads in one of the samples) and (3) decreased in the PostOp EVs as compared to PreOp EVs (LogFC>1, adj. *p* < 0.05). RNAs matching these criteria were found in four RNA biotypes: miRNA, piRNA, mRNA and lncRNA (Figures 3, 4).

A total of 445 different miRNAs were found in small RNA libraries generated from PC and normal prostate tissues and 54 of them were overexpressed in tumor, whereas 382 distinct miRNAs were present in urinary EVs and 331 in plasma EVs (Figure 3A). However, the levels of only 3 miRNAs were significantly decreased in the PostOpU EVs as compared to the PreOpU EVs and two of them - miR375-3p and miR92a-1-5p were overexpressed in tumor tissue (Figure 3A). No such candidates were found in plasma EVs.

A total of 264, 298 and 221 piRNAs were found in the tissues, urinary and plasma EVs, respectively (Figure 3B). The levels of 9 and 1 piRNA were decreased in the PostOpU and PostOpP EVs, respectively, however only one of them—piR-28004, was also overexpressed in PC tissues (Figure 3B).

To identify mRNAs and lncRNAs that are overexpressed in PC, full transcriptome libraries were analyzed. Differential expression analysis revealed 4401 mRNA and 2864 lncRNAs overexpressed in cancer as compared to normal tissues. The levels of 139 mRNAs were decreased in the PostOpU EVs and 63 of them overlapped with those overexpressed in PC tissues, thus showing that mRNAs are the most abundant type of cancer-derived RNA biomarkers in urinary EVs. At the same time, only one mRNA was found to be decreased in the PostOpP EVs and it was not significantly overexpressed in PC. The levels of 16 lncRNAs were decreased in urinary EVs after surgery; 3 of them–Linc00662, CHASERR and lnc-LTBP3-11 were overexpressed in PC tissues and represent the PC biomarker candidates.

**FIGURE 5:**
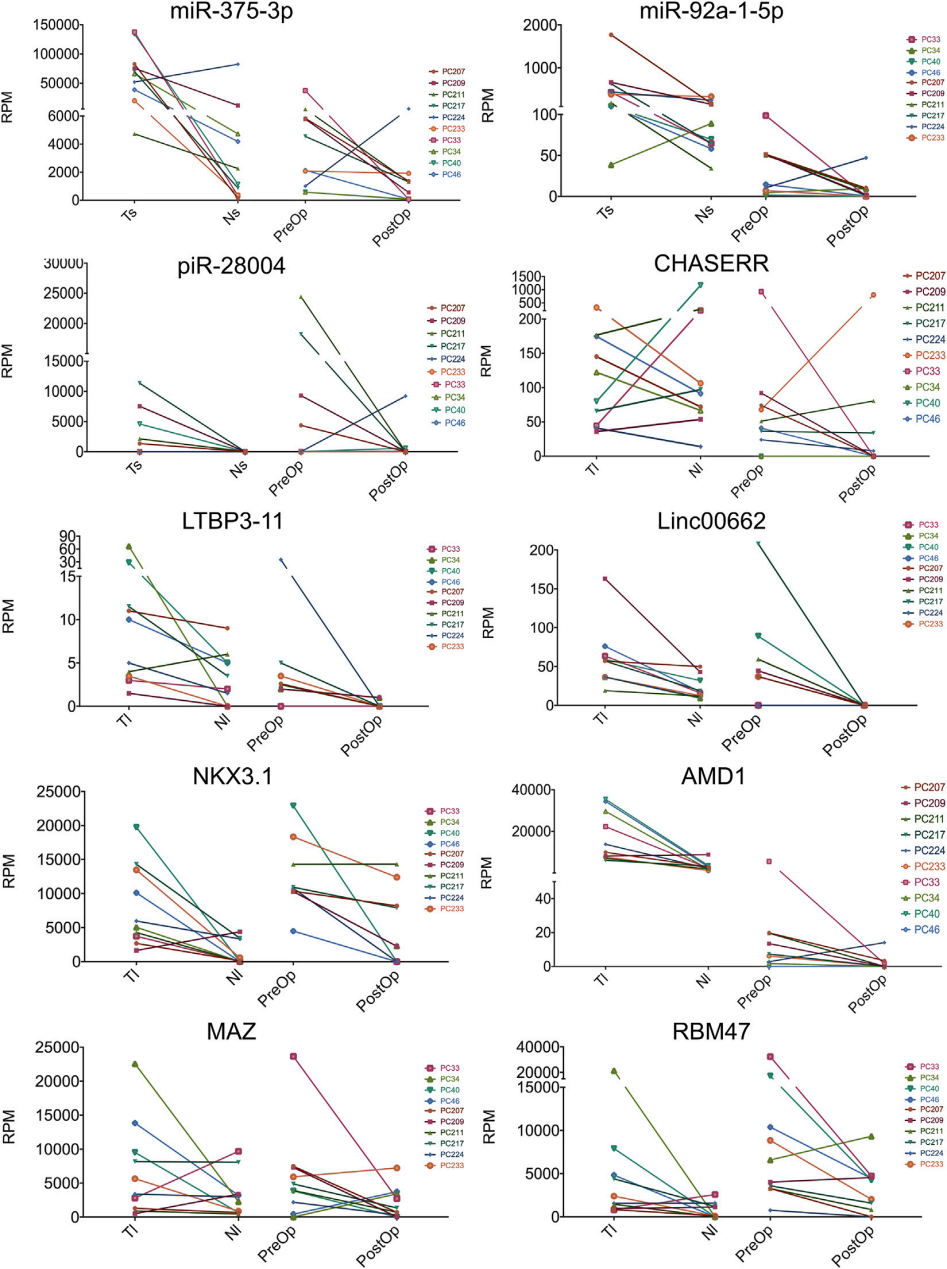
Selected biomarker candidates. The paired dot plots show the normalized read counts (reads per million mapped reads, RPM) for selected RNA biomarkers in tumor and normal prostate tissue small RNA libraries (TS and NS, respectively) or full transcriptome libraries (TL and NL), and pre-operation and post-operation urinary EVs. Log2 fold changes and multiple testing adjusted *p* values are shown in Supplementary Table S2.

Taking together, a total of 63 mRNAs, 3 lncRNAs, 2 miRNAs and 1 piRNA were identified as potential PC-derived biomarker candidates (Figure 5; Supplementary Table S2).

### Validation of selected PC biomarkers by RT-ddPCR

Based on the expression levels, fold changes and functional significance, a set of biomarker candidates representing various RNA biotypes was selected. For each of the candidates, LNA-based primers (QuantiNova LNA PCR custom assays or miRCURY LNA miRNA PCR assays depending on the target length and RNA biotype) were designed using GeneGlobe (Qiagen) platform. As sequencing data suggested that the EV-enclosed mRNAs and lncRNAs are fragmented, we selected target regions that were present in the majority of the EV samples. Using this approached we failed to identify suitable target regions for all three lncRNAs and several mRNAs because the fragments were too short or had unacceptably high GC content. Nevertheless, seven RT-ddPCR assays (miR-375-3p, hasa-piR-28004, GLO1, NKX3-1, AMD1, MAZ and RBM47) were successfully designed (Supplementary Table S1) and their performance was validated by testing the same PC and normal prostate specimens that were used for RNA sequencing analysis. Next, these assays were used to analyze the levels of candidate biomarkers in an independent, longitudinal cohort of PreOp and PostOp urinary and plasma EV samples from 20 patients with PC.

**FIGURE 6:**
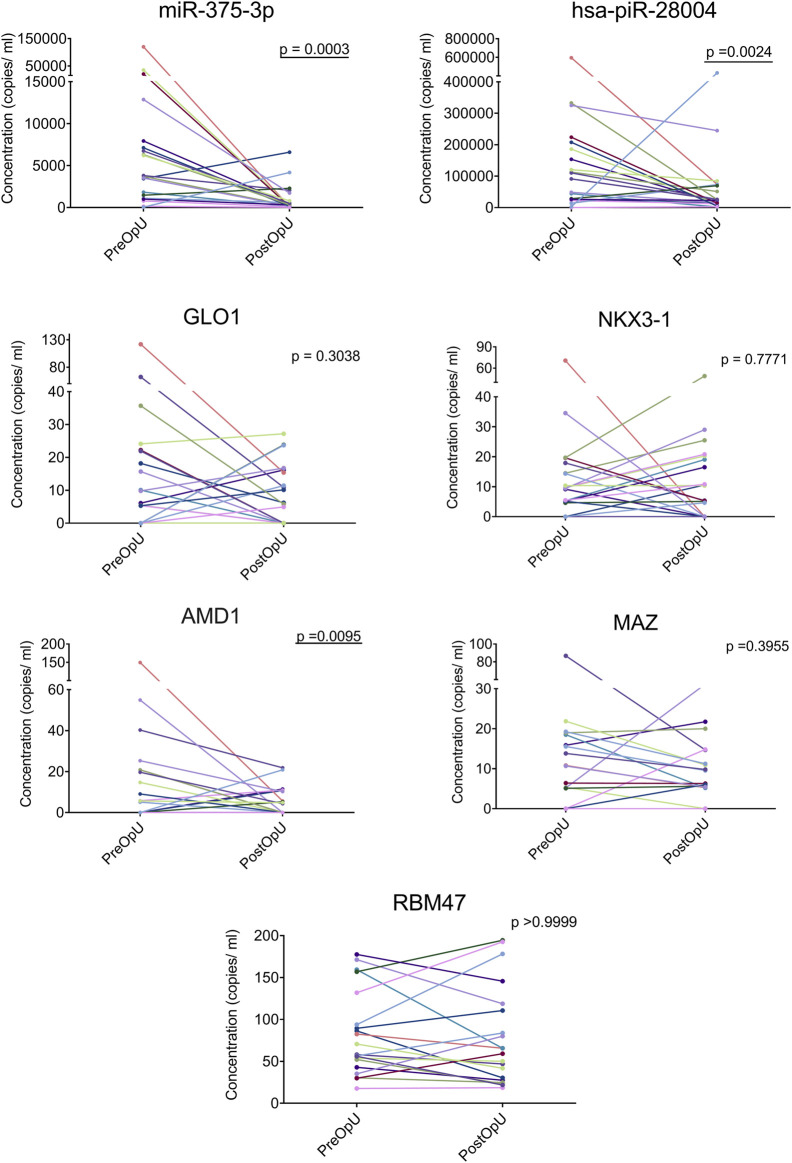
Validation of selected biomarker candidates in urinary EVs from an independent cohort of 20 PC patients by RT-ddPCR. Paired dot plots show the copy number of RNA biomarkers per ml of urine collected before and after radical prostatectomy in 20 PC patients. Wilcoxon matched-paired signed rank test was used to assess the statistical significance of the differences between groups. *p*-value < 0.05 was considered significant.

In urinary EVs, the levels of miR-375-3p (FC = 11.49; *p* = 0.0003), piR-28004 (FC = 2.18, *p* = 0.0024) and adenosylmethionine decarboxylase 1 (AMD1) (FC = 3.49, *p* = 0.0095) were significantly decreased in the PostOp as compared to the PreOp samples (Figure 6). The levels of GLO1, MAZ and NKX3-1 mRNAs were also decreased in a fraction of patients, yet didn’t reach statistical significance. None of these biomarker candidates was significantly altered in the PostOp plasma EVs (Supplementary Figure S1).

## Discussion

Several earlier studies have shown that patients with various cancers have higher levels of EVs in the circulation than cancer-free controls (Logozzi et al., 2009; Ogorevc et al., 2013; Alegre et al., 2016; Matsumoto et al., 2016; Rodriguez Zorrilla et al., 2019) and suggested that increased EV levels are associated with disease progression and therapy failure (Konig et al., 2017), thus leading to the idea that the EV level could be a cancer biomarker on its own. We, however, didn’t observe consistent and significant decrease in the plasma and urinary EV levels in PC patients 3 months after the surgery, suggesting that the presence of PC doesn’t substantially affect the total level of EVs neither in plasma nor urine. There are several technical issues that may lead to controversial results on the EV counts. At first, the current EV quantification methods require pre-isolation of EVs from biofluids. EV isolation methods, in turn, differ in their efficiency, EV size range and the level of lipoprotein co-isolation that may affect the NTA measurements. Therefore, novel technologies that would allow quantification of EVs directly in biofluids are needed for accurate assessment of EV levels. Alternatively, it is possible that the prostate, which, on average, is about the size of a walnut and weights around 20–25 grams, can’t substantially contribute to the total pool of EVs and even if the EV production is increased in the cancerous tissues, this would not significantly alter the total EV levels. Since it has been shown that prostate massage can induce the secretion of prostatic fluid into the urethra that in turn increases the fraction of prostate-derived EVs in urine (Duijvesz et al., 2015), it might be beneficial to perform the prostate massage before collecting the urine samples for EV analysis, however, it would make the test less amenable for routine applications.

Since the discovery of cancer-derived RNA molecules in EVs isolated from biofluids of cancer patients, EVs have gained a considerable interest as a source of RNA biomarkers for liquid biopsies of cancer (Vasconcelos et al., 2019). So far, most of the studies investigating the EV-enclosed RNA cargo in PC patients have been designed as case-control studies (Rodríguez et al., 2017; He et al., 2021; Ramirez-Garrastacho et al., 2022a; Ramirez-Garrastacho et al., 2022b). Although these studies have revealed a number of potential PC biomarkers, the cellular origin of these biomarkers remained unknown. In the current study, we took a different, patient-centered approach to characterize plasma and urinary EVs as carriers of PC-derived RNAs. We reasoned that combining three criteria: overexpression in tumor tissues, high abundance in PreOp EVs and decrease in the PostOp EVs would allow to identify RNA biomarkers that are derived from PC tissues.

This approach apparently was successful for the identification of PC-derived RNAs in urinary EVs–a total of 69 biomarker candidates representing four RNA biotypes were identified. Many of the protein coding genes have been previously shown to be overexpressed in PC and functionally implicated in the development or progression of PC thus supporting their use as PC biomarkers. For instance, FOXA1, NWD1 and MAZ have been shown to promote tumor progression by modulation of androgen receptor expression and are frequently linked with poor prognosis (Gerhardt et al., 2012; Jiao et al., 2013; Correa et al., 2014; Parolia et al., 2019). In addition, MAZ has been reported to promote bone metastasis in PC cells by transcriptionally activating RAS signaling pathway (Yang et al., 2019). LCP1 was correlated with lymph node metastasis and proposed as independent PC prognostic factor (Chen et al., 2017), while GLO1 might serve as a high-grade prostatic intraepithelial neoplasia marker (Rounds et al., 2021) and is strongly linked to early biochemical recurrence (Burdelski et al., 2017). Similarly, TGM4 has been shown to induce EMT transition in PC cells (Ablin et al., 2017) and its overexpression has been linked to poorer outcomes (Cao et al., 2013). Interestingly, TGM4 as well as NKX3.1 have been identified as prostate-restricted markers (Gurel et al., 2010; Lopez-Bujanda et al., 2021).

To the best of our knowledge, none of these mRNAs has been previously reported to be present in EVs. On the contrary, miR-375-3p was found to be present in serum, plasma and urinary EVs of PC patients in multiple studies and its level could distinguish patients with PC from patients with BPH and healthy men as well as correlate with the disease outcome (Endzelins et al., 2016; Endzeliņš et al., 2017; Ramirez-Garrastacho et al., 2022a).

lncRNA Linc00662 has been shown to promote tumorigenesis in PC (Yao et al., 2020), CHASERR, an evolutionary conserved lncRNA, regulates the levels of chromatin remodeling protein CHD2 (Antonov and Medvedeva, 2020), whereas the role of the third lncRNA identified in this study is unknown. None of them have been described as EV-enclosed PC biomarker previously. So far, the most well studied EV-enclosed lncRNA is PCA3, which has been shown to differentiate between PC patients and healthy males as well as between PC patients with GS ≤ 6 and GS ≥ 7 tumors (Ramirez-Garrastacho et al., 2022a) and is included in the FDA approved ExoDx PC test that informs whether to proceed with prostate biopsy in men with a PSA levels between 2 and 10 ng/mL (Tutrone et al., 2020). In our study, PCA3 was highly overexpressed in PC tissues (LogFC = 9.08, adj. *p* = 1.32 × 10^−85^), present in PreOpU EVs and decreased after the surgery, yet the difference did not reach statistical significance after multiple testing correction.

Among the biomarker candidates was also piR-28004, an uncharacterized piRNA, whereas the levels of another 8 and 1 piRNA were decreased in the PostOp U and PostOpP EVs, respectively and another 10 were significantly overexpressed in PC tissues. piRNAs are 26–32 nt long RNA molecules that interact with PIWI proteins and act as mediators of various processes including transposon silencing, genome rearrangement, epigenetic regulation of gene expression etc. Initially, piRNAs were thought to function mainly in male germ cells, however it is now clear that they are aberrantly expressed in multiple types of cancer and play essential roles in the progression of cancer and metastasis by transcriptional and post-transcriptional gene silencing (Wu et al., 2020; Qian et al., 2021; Riquelme et al., 2021). Recently, a number of piRNAs were found in urinary EVs of PC patients and the levels of four of them differed between PC patients and healthy controls (Peng et al., 2021). Given that more than 30 000 piRNAs have been discovered in the genome and their widespread deregulation in cancers, they appear to be a very rich source of cancer biomarkers. However, currently their analysis is hampered by poor annotation in the human genome and inconsistent data and nomenclature in the piRNA sequence databases such as piRBase (Wang et al., 2018), piRNABank (Sai Lakshmi and Agrawal, 2008) and piRNAdb (Piuco and Galante, 2021).

The most straight forward way to translate these findings into clinically applicable tools, would be to develop PCR-based assays, however, this is hampered by two major issues: fragmentation of long RNAs and the lack of normalization methods for urinary EV analysis. Long RNAs such as mRNAs and lncRNAs appear to be an attractive type of biomarkers due to higher fold changes, known functional significance in the cancer and considerably higher number of biomarker candidates as to compare with miRNAs or other small non-coding RNAs. However, the design of PCR assays for long RNAs is challenging due to their fragmented nature in EVs (Hulstaert et al., 2020). Currently, it is not known if the fragmentation pattern of RNAs is entirely random and if some of the fragments are preferentially included or excluded from EVs, and what is the variation in the sorting of RNA fragments into EVs among different individuals.

To validate RNA sequencing data that are normalized for sequencing depth and RNA composition, PCR normalization method that is based on a set of invariable reference genes is needed. Some potential reference genes have been identified for blood EVs (Gouin et al., 2017; Dai et al., 2021; Damanti et al., 2021), but not for urinary EVs. Moreover, majority of the candidate genes are miRNAs that most likely are not suitable for other RNA biotypes and so far they have not been validated by independent studies. In the current study, we normalized the RT-ddPCR results to the volume of biofluids. For plasma, it is widely acceptable way of normalization (van Eijndhoven et al., 2016; Endzeliņš et al., 2017), but it is not the best option for urinary EVs since the volume and concentration of urine can vary substantially. Several normalization methods for urinary biomarker studies have been proposed, such as urinary creatinine level or total EV number (Erdbrugger et al., 2021), however, they have several limitations and/or may be problematic for the analysis of prostate-derived EVs. Creatinine is a waste product of muscle catabolism and its levels have been shown to vary between individuals due to the age, physical activity, muscle mass, diet, stress etc. (Tang et al., 2015). Furthermore, the levels of prostate-derived EVs are unlikely to be linearly related to creatinine excretion rate. In turn, EV number is highly dependent on the EV isolation and quantification method that may introduce a large bias. Moreover, it might be problematic in case the increased EV production is a part of the disease pathophysiology. More recently, urinary PSA level has been proposed as a normalizer specifically for studying prostate-derived EVs (Duijvesz et al., 2015), however it requires further validation in urinary EVs (Erdbrugger et al., 2021). Hence, currently, the sequencing-based methods appear to be more reliable tools for the analysis of EV-RNAs.

Taken together, these data suggest that the fraction of PC-derived EVs in urine is sufficiently large to allow the detection and tracking of PC-derived RNAs. Hence, urine appears to be a superior source of EV-RNAs for the diagnosis and active surveillance of PC, however, it is unlikely to be suitable for post-operative monitoring of PC progression. For this, blood assays are needed. However, our data suggest that the PC-derived EV fraction in blood plasma at least in patients with localized PC is too low to enable tracking of PC-derived RNAs. Moreover, the composition of plasma EVs is much more complex than urinary EVs as EVs are sampled from many more different cell types, hence EV isolation methods that would allow enrichment with cancer-derived EVs are required for the development of EV-based blood tests for the detection and monitoring of PC.

## Data Availability

The datasets presented in this study can be found in online repositories. The names of the repository/repositories and accession number(s) can be found in the article/Supplementary Material.

## Supplementary material

SUPPLEMENTARY FIGURE S1 Testing of selected biomarker candidates in plasma EVs from an independent cohort of 20 PC patients by RT-ddPCR. Paired dot plots show the copy number of RNA biomarkers per ml of plasma collected before and after radical prostatectomy in 20 PC patients. Wilcoxon matched-paired signed rank test was used to assess the statistical significance of the differences between groups. *p*-value < 0.05 was considered significant.

## Abbreviations

DEG, differentially expressed gene; EVs, extracellular vesicles; lncRNA, long non-coding RNA; miRNA, microRNA; miscRNA, miscellaneous RNA; mRNA, messenger RNA; MT-RNA, mitochondrial RNA; NTA, nanoparticle tracking analysis; PC, prostate cancer; piRNA, piwi-interacting RNA; PreOp, Pre-Operation; PostOp, Post-Operation; RP, Radical Prostatectomy; rRNA, ribosomal RNA; SEC, Size Exclusion Chromatography; snRNAs, small nuclear RNAs; snoRNAs, small nucleolar RNAs; TEM, transmission electron microscopy; tRNA, transfer RNA; WB, Western Blot.
