# Supplementary figures and images for: Plasma and urinary extracellular vesicles as a source of RNA biomarkers for prostate cancer in liquid biopsies
Source: Front Mol Biosci. 2023 Feb 3;10:980433. doi: 10.3389/fmolb.2023.980433 (PMC9935579; doi:10.3389/fmolb.2023.980433)

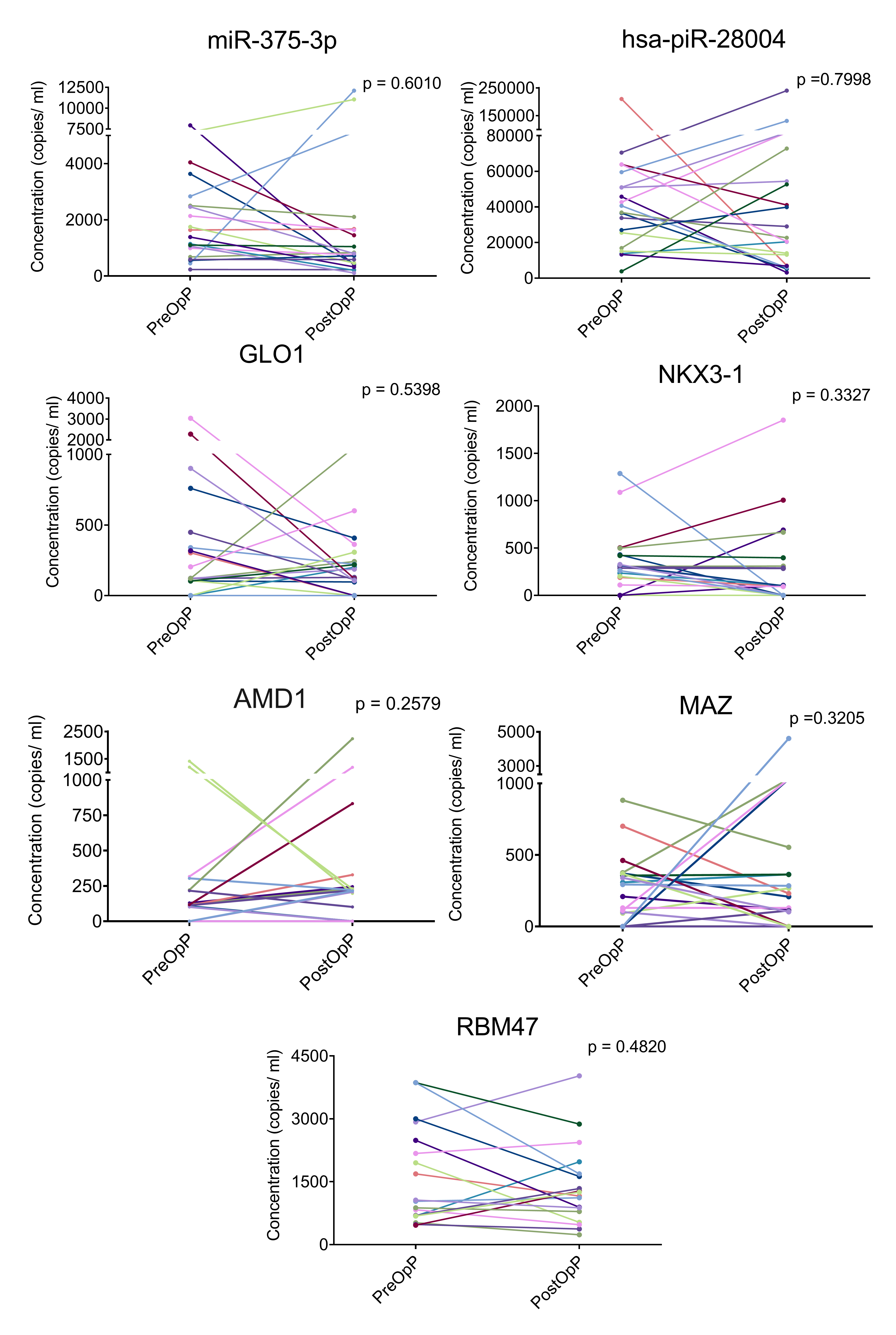

Supplement: Supplementary file 2 [file Image1.TIFF]
