# Supplementary material for: Plasma and urinary extracellular vesicles as a source of RNA biomarkers for prostate cancer in liquid biopsies
Source: Front Mol Biosci. 2023 Feb 3;10:980433. doi: 10.3389/fmolb.2023.980433 (PMC9935579; doi:10.3389/fmolb.2023.980433)
Supplement: Supplementary file 3 [file Table1.DOCX]

**Table S1.** ddPCR primer assays.

| **Gene name** | **Assay Type** | **Accession number*** | **Annealing temperature** | **Target sequence** |
| --- | --- | --- | --- | --- |
| miR-375-3p | miRCURY LNA | YCP1552280 | 55ºC | UUUGUUCGUUCGGCUCGCGUGA |
| hsa-piR-28004 | miRCURY LNA | YCP1551995 | 54ºC | GCATTGGTGGTTCAGTGGTAGAATTCTCGCCT |
| GLO1 | QuantiNova LNA | SCB0419367 | 54ºC | TGAAGTGATGAGACCCAGAGTTACCA |
| NKX3-1 | QuantiNova LNA | SCB0419359 | 56ºC | AAAGAGGAGGCCTTCTCCCGGGC |
| AMD1 | QuantiNova LNA | SCB0419381 | 55ºC | GTCTCACGGTGATGGAAGCTGCACATTTTTTCGAAGGGACCGAGAAGCTGCTGGAGGTTTGGT |
| MAZ | QuantiNova LNA | SCB0420996 | 54ºC | ATGAAGGTGCACAGCCAGGGTCCTCACCATGTCTGTGAGCTCTGCAACAAAG |
| RBM47 | QuantiNova LNA | SCB0412069 | 56ºC | AAGAAAAAGGATATGAACTGGTGCCGAA |

*Accession numbers corresponding to GeneGlobe platform (Qiagen).
